# CSF-1 Receptor-Dependent Colon Development, Homeostasis and Inflammatory Stress Response

**DOI:** 10.1371/journal.pone.0056951

**Published:** 2013-02-22

**Authors:** Duy Huynh, Dilara Akçora, Jordane Malaterre, Chee Kai Chan, Xu-Ming Dai, Ivan Bertoncello, E. Richard Stanley, Robert G. Ramsay

**Affiliations:** 1 Peter MacCallum Cancer Centre, East Melbourne, Victoria, Australia; 2 Department of Genetics, Latrobe University, Victoria, Australia; 3 Department of Pathology, The University of Melbourne, Parkville, Victoria, Australia; 4 Sir Peter MacCallum Department of Oncology, The University of Melbourne, Parkville, Victoria, Australia; 5 Department of Developmental and Molecular Biology, Albert Einstein College of Medicine, New York, New York, United States of America; 6 Department of Pharmacology the University of Melbourne, Parkville, Victoria, Australia; National Institute of Agronomic Research, France

## Abstract

The colony stimulating factor-1 (CSF-1) receptor (CSF-1R) directly regulates the development of Paneth cells (PC) and influences proliferation and cell fate in the small intestine (SI). In the present study, we have examined the role of CSF-1 and the CSF-1R in the large intestine, which lacks PC, in the steady state and in response to acute inflammation induced by dextran sulfate sodium (DSS). As previously shown in mouse, immunohistochemical (IHC) analysis of CSF-1R expression showed that the receptor is baso-laterally expressed on epithelial cells of human colonic crypts, indicating that this expression pattern is shared between species. Colons from *Csf1r* null and *Csf1^op/op^* mice were isolated and sectioned for IHC identification of enterocytes, enteroendocrine cells, goblet cells and proliferating cells. Both *Csf1r^−/−^* and *Csf1^op/op^* mice were found to have colon defects in enterocytes and enteroendocrine cell fate, with excessive goblet cell staining and reduced cell proliferation. In addition, the gene expression profiles of the cell cycle genes, *cyclinD1*, *c-myc*, *c-fos*, and *c-myb* were suppressed in *Csf1r^−/−^* colonic crypt, compared with those of WT mice and the expression of the stem cell marker gene *Lgr5* was markedly reduced. However, analysis of the proliferative responses of immortalized mouse colon epithelial cells (lines; Immorto-5 and YAMC) indicated that CSF-1R is not a major regulator of colonocyte proliferation and that its effects on proliferation are indirect. In an examination of the acute inflammatory response, *Csf1r* ^+/−^ male mice were protected from the adverse affects of DSS-induced colitis compared with WT mice, while *Csf1r* ^+/−^ female mice were significantly less protected. These data indicate that CSF-1R signaling plays an important role in colon homeostasis and stem cell gene expression but that the receptor exacerbates the response to inflammatory challenge in male mice.

## Introduction

Colonic and small intestinal (SI) crypts share a range of architectural and molecular features and for convenience are often discussed interchangeably. Each generates the three cell lineages; the enterocytes, which secrete hydrolases and absorb nutrients, [1] mucin-producing goblet cells [2], [3] and the less common enteroendocrine cells that secrete a spectrum of mediators including serotonin, secretin and substance P [4], [5]. Similarly, both crypt compartments are regulated by Wnt signaling and dysregulation of this pathway initiates adenoma formation [6]. However, there are as several important differences. The SI possesses PC, which reside as a cluster of up to 16 cells at the SI crypt base [7] that out live the cells of the other three SI lineages and are absent in the colonic crypt. In addition, the SI contain villi, absorptive structures (predominantly enterocytes) supported by approximately 6 crypts, that increase the epithelial surface area of the SI [8].

We have recently shown that the cytokine colony stimulating factor receptor (CSF-1R) is required for PC development and that CSF-1R-deficient mice possess deficits in SI enterocytes and enteroendocrine cells and excess goblet cell production, as well as substantively reduced crypt proliferative capacity and stem cell niche maintenance [9]. Here we demonstrate a role for CSF-1R in the colon, thereby uncoupling the CSF-1R-dependence of PC from the effects of CSF-1R on enterocyte proliferation and lineage specification.

*In vivo* CSF-1 regulation has been investigated in the CSF-1-deficient *osteopetrotic (Csf1^op/op^*) mutant mouse, [10] which possesses an inactivating mutation in the *Csf1* gene [11], [12], [13], [14] and in CSF-1R-deficient *Csf1r^−/−^* mice with a targeted deletion of the only cellular receptor for CSF-1 [15], [16]. These studies unequivocally established that CSF-1 is the primary regulator of macrophage, [17] osteoclast [16] and Langerhans [18] cell production via CSF-1R signaling [15]. Using approaches pioneered by investigators of the hematopoietic system, we previously showed that CSF-1 supported the colony formation of fetal and new born colonocytes *in vitro* and that CSF-1R is expressed on the basal lateral surface of mouse colonic crypts [19]. In parallel with the defects we have reported in the SI of mice with defective CSF-1R signaling [9], we now show that these mice also have proliferative deficits in the colonic crypts. Furthermore, the expression of the intestinal stem cell marker gene, Lgr5 and proliferation-associated genes indicative of progenitor cell activity are also reduced. In addition, using an established model for inflammatory bowel disease, we show that heterozygous loss of the *Csf1r* gene is protective in male mice. Collectively, these studies support a central role for CSF-1R signaling in gastrointestinal homeostasis and disease.

## Results

### The CSF-1R is Expressed in Human Colonic Crypts

**Figure 1:**
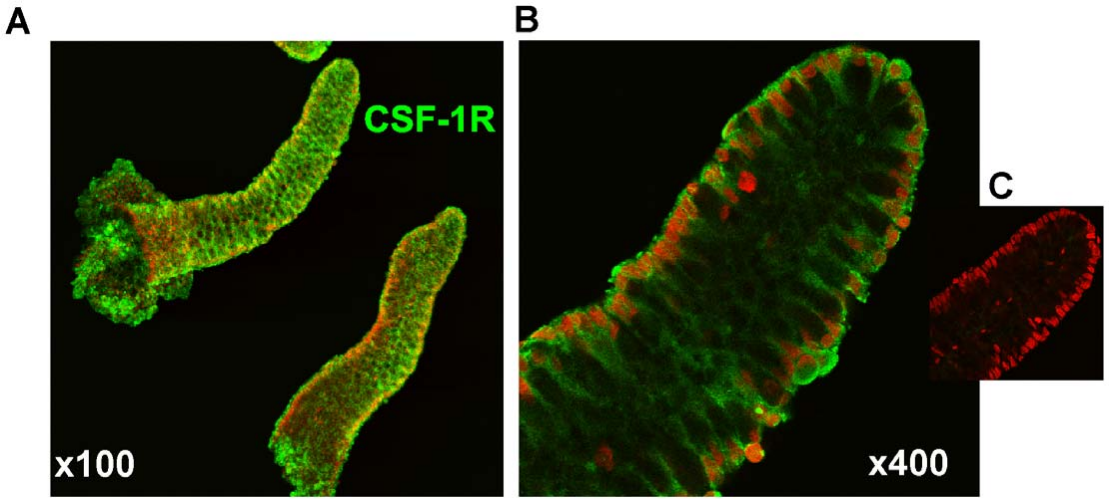
Isolated human colonic crypts display robust expression of cell surface CSF-1R. (A) Low power and (**B**) high power confocal images stained with FITC-conjugated anti-CSF-1R antibody (green) or (**C**) secondary antibody alone and counter-stained with propidium iodine (red).

We have previously reported the baso-lateral presence of CSF-1R on isolated mouse colonic crypts [19]. When human crypts were similarly examined using a human CSF-1R-specific antibody and counter-stained with propidium iodide essentially identical images were generated (**Figure 1**). These data and our previous report of SI defects in mice with ablated CSF-1R signaling prompted us to investigate the role of the CSF-1R in the colon.

### Altered Colon Metrics and Cell Fate in Colons of CSF-1- and CSF-R- Deficient Mice

**Figure 2:**
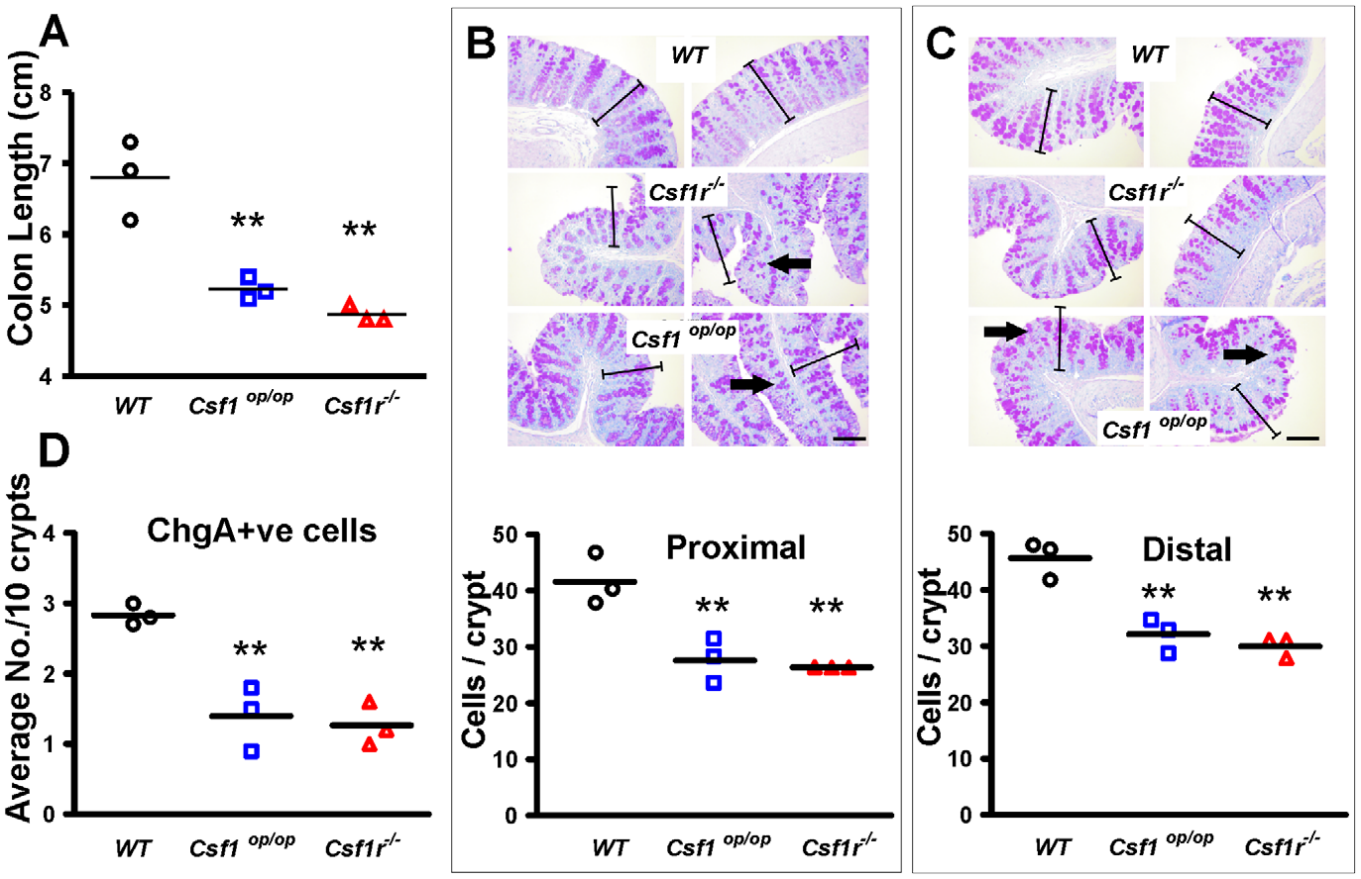
Two-week old *Csf1r^−/−^* and *Csf1^op/op^* mutant mice have shorter colons, fewer cells per crypt and fewer enteroendocrine cells. (A) Colon lengths for each genotype are represented (n = 3). (**B,C**) Colon sections from proximal (**B**) and distal (**C**) regions were stained with PAS to highlight neutral mucin production. The crypt length bars defined in WT colons (**B**) were duplicated and superimposed on the images in (**C**) to illustrate the shortened crypts of the mutant mice. Bars = 50 µm. Lower panels show the number of cells per crypt cross section. (**D**) Average numbers of enteroendocrine cells as visualized by Chromogranin A immunohistochemistry are plotted. Means are depicted by horizontal bars, n = 3 per group, (**P<0.01 using unpaired two-tailed t-tests).

Colons of two-week old *Csf1r^−/−^* and *Csf1^op/op^* mutant mice and matching wild type (WT) littermate controls were excised from cecum to anus. Three mice of each genotype were evaluated and both mutants were found to have significantly shorter colons (p>0.01), commensurate with their reduced overall size (**Figure 2A**). Tissue was processed for cytochemistry and IHC. Proximal and distal colon regions were evaluated separately. In both regions it was evident that the mucosal thickness in mutant mice was less than that of WT mice (**Figure 2B–C**). Enumeration of cell nuclei in definable crypts revealed that the number of cells per crypt was significantly less in mutants compared to WT (**Figure 2B–C**). PAS (Periodic Acid Schiff) staining suggested that the degree of mucin staining was greater in sections from the mutant mice (**Figure 2B–C**). To explore this observation further, section were also stained with Alcian Blue and the level of mucin staining in the mutant colons was consistently observed to be greater than in WT colons (**Figure S1**). Staining of sections for Chromogranin A, to evaluate the number of enteroendocrine cells for the whole colon, revealed that both mutant mice had fewer enteroendocrine cells than WT (p<0.01) (**Figure 2D**). These data indicate that the mutant mice have shorter colons, with fewer enterocytes (the predominant cell lineage in the crypt), fewer enteroendocrine cells and greater mucin production.

### Defective Cell Proliferation in *Csf1r ^−/−^* and *Csf1^op/op^* Colonic Epithelia

**Figure 3:**
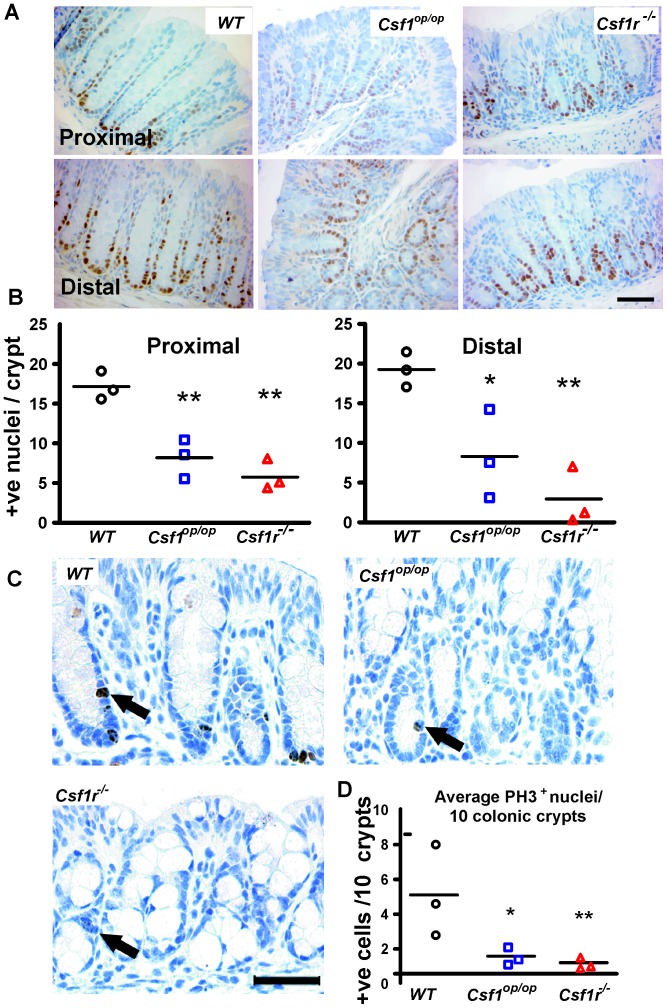
Decreased proliferation in *Csf1r^−/−^* and *Csf1^op/op^* mouse colonic epithelium. (**A**) Representative PCNA immunostaining in proximal and distal colon sections are shown. (**B**) Enumeration of the total number of positively stained nuclei reveals that average number of proliferating cells/crypt is significantly reduced in proximal (left panel) and distal (right panel) colons of *Csf1^op/op^* and *Csf1r^−/−^,* compared to WT, mice. Bar = 50 µm (n = 3). (**C**) Staining with the G_2_/M phase marker, phosphorylated histone H3 (PH-3, black arrows) also shows decreased numbers of cells in cycle per crypt in both mutants. Bar = 50 µm. Means are depicted by horizontal bars, n = 3 per group, **P<0.01, *P<0.05, using unpaired two-tailed t-test. (**D**) Quantitation the PH-3^+^ staining where means are depicted by horizontal bars, (n = 3 per group). Having predicted a reduced level of PH3 in *Csf1r^−/−^* crypts data was analyzed using one-tailed t-tests, (**P<0.01, *P<0.05).

In view of the shorter crypts in the mutant mice, we postulated that proliferation within their crypts was defective. To measure this, sections were stained for proliferating cell nuclear antigen (PCNA). Images of these sections (**Figure 3A**) suggesting that there are fewer positive cells in the mutant crypts was confirmed by counting positive nuclei per crypt (proximal; P<0.01), (distal; *Csf1^op/op^*; P<0.05, *Csf1r^−/−^*; P<0.01) (**Figure 3B**). PCNA identifies cells that are in cell cycle but does not provide information about whether cells are progressing through the cell cycle. Accordingly, sections were additionally stained for the G_2_/M marker, phospho-histone 3 (PH3) which showed that the mutant crypts had fewer cells that had progressed to these later phases of the cell cycle (distal plus proximal: *Csf1^op/op^*; P<0.05, *Csf1r^−/−^*; P<0.01) (**Figure 3C–D**).

### Reduced Expression of Cell Cycle Genes in *Csf1r ^−/−^* Crypt Epithelial Cells

**Figure 4:**
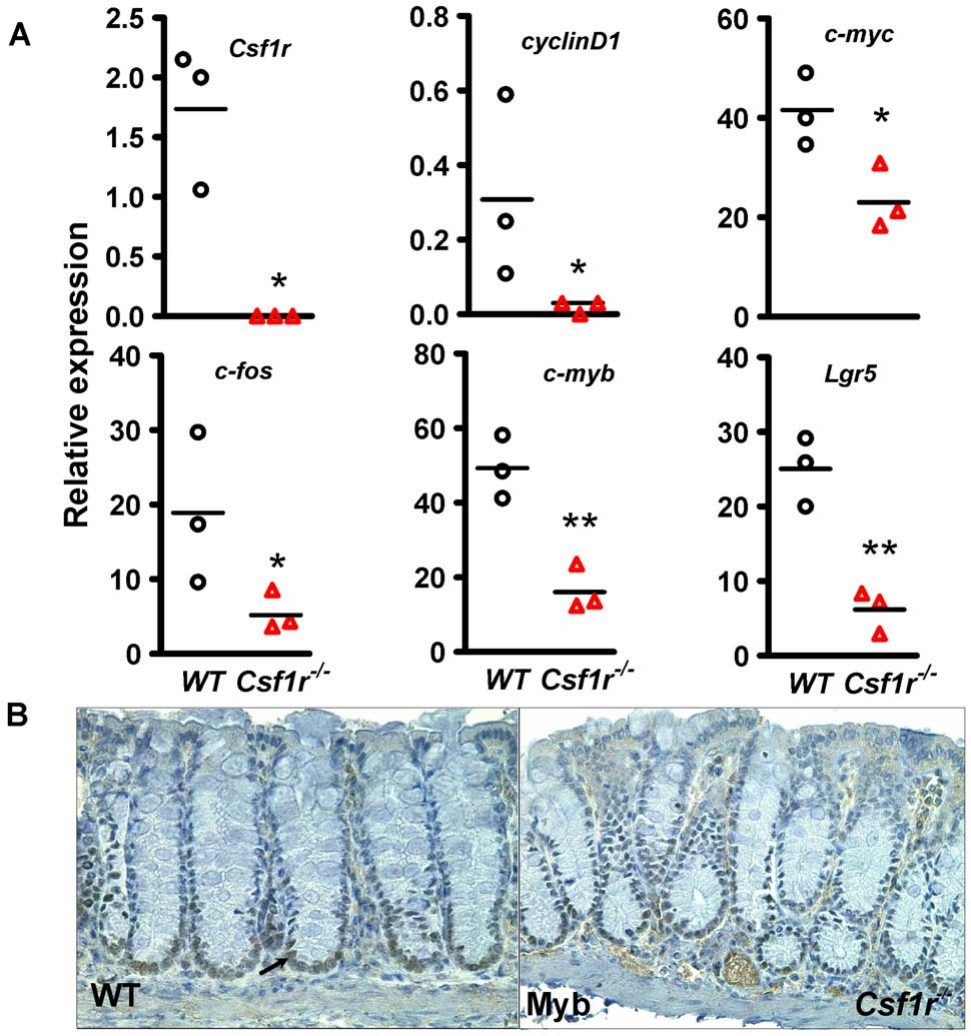
*Csf1r^−/−^* colonic crypts show reduced expression of cell cycle gene mRNAs compared to WT colonic crypts. (**A**) RNA from WT and *Csf1r^−/−^* crypts was subjected to qRT-PCR analysis for expression of *Csf1r,* the cell cycle genes *cyclinD1 and c-myc, c-myb* and the immediate early gene, *c-fos,* as well as the intestinal stem cell marker, *Lgr5.* Expression of all six genes was reduced in the *Csf1r^−/−^* compared with WT crypts, (n = 3, *P<0.05; **0.01). (**B**) Immunohistochemical staining for Myb. In addition to a shorter crypt length, the expression of Myb, which has a direct effect on this metric [23], is less extensive in *Csf1r^−/−^* crypts. Black arrow indicates Myb positive nucleus. Means are depicted by horizontal bars, n = 3 per group and having predicted a reduced level of gene expression in *Csf1rKO* crypts data were analyzed using one-tailed t-tests**P<0.01, *P<0.05.

As the defect in proliferation was slightly worse in *Csf1r ^−/−^* mice, we focused attention on colons from these mice to investigate the expression of genes involved in cell cycle progression. Colonic crypts from WT and *Csf1r^−/−^* mice were isolated for qRT-PCR analysis. As expected *Csf1r* expression was undetectable in the *Csf1r^−/−^* mice. The expression of growth factor receptor target genes, *c-myc* and *cyclinD1* [20], [21] and the immediate early response target gene *c-fos* [22] were expressed at lower levels (P<0.05) in the colonic epithelium *of Csf1r^−/−^* compared to WT mice (**Figure 4**). As we had previously reported a role for *c-myb* in colon homeostasis [23] this gene was also evaluated and found to be expressed at a lower level (P<0.003). Finally, the intestinal stem/progenitor marker *Lgr5* [24] which we have found to be in part regulated by Myb [25] was similarly found to be significantly under-expressed (P<0.002) (**Figure 4A**). Immunochemical staining of Myb (**Figure 4B**) confirmed that Myb protein expression was lower in the *Csf1r^−/−^* mice. This evaluation of genes associated with proliferation confirms that the reduced proliferation found in the *Csf1r^−/−^* colonic crypts and their correspondingly shorter length is correlated with reduced cell cycle gene expression. These data also highlight the importance of the CSF-1R in maintaining intestinal stem cell gene expression.

### Modest Proliferation Colonic Epithelial Cells in Response to CSF-1R Ligands *in vitro* is Consistent with Indirect Regulation of Epithelial Cell Proliferation by these Ligands *in vivo*

To further explore the role of the CSF-1R signaling in colonic epithelial cells, we examined the response of cells of the immortalized, colonic epithelial cell lines YAMC [26] and Immorto-5 [19] to growth factor stimulation following serum starvation. Under these conditions, pregnant mouse uterus extract (PMUE), a good source of CSF-1 [19], [27], [28] stimulated robust YAMC growth (**Figure S2A**), phospho-ERK1/2 induction (**Figure S2B**) and *c-myb*, *c-myc*, *cyclinD1*, *Ets-2* and *c-jun* gene expression (**Figure S3**). Cytokine antibody arrays confirmed the presence of CSF-1 in PMUE, but also demonstrated the presence of several other factors (**Figure S2C**). However, attempts to replicate the level of growth stimulation with either purified recombinant CSF-1, or purified IL-34, which also activates the CSF-1R [29], were only partially successful (**Figure S2D–E**). Although these experiments were carried out with cell lines, the lack of a strong proliferative response to CSF-1R ligands raised the possibility that the *in vivo* requirement of the CSF-1R for epithelial cell proliferation is indirect. As recent studies have shown that PC, the CSF-1R-responsive cells of the SI [9], produce two key epithelial cytokines Wnt3 and R-Spondin [30], we tested the ability of these factors to stimulate proliferation of our colonic epithelial cells. Consistent with an indirect effect of CSF-1 on epithelial cell proliferation, robust cell proliferation was seen with R-spondin, with or without CSF-1 plus Wnt3a, but not with Wnt3a alone (**Figure S2F**).

### Loss of a Single *Csf1r* Allele in Male Mice Alleviates DSS-induced Colitis

**Figure 5:**
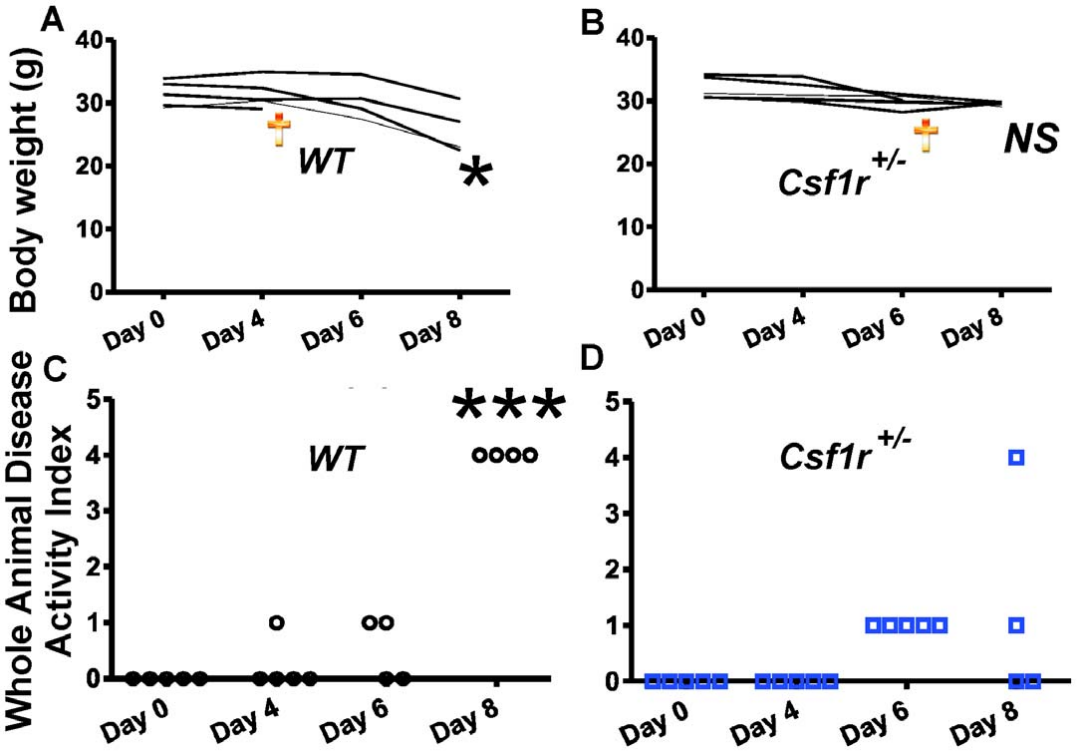
Loss of one *Csf1r* allele in male mice is protective for weight loss and symptoms associated with DSS-induced colitis. Eight to 10-week old FVB/NJ male mice were given water with 2% w/v DSS *ad libitum* for 8 days and their body weight changes monitored during this period. (**A–B**) Significant changes in body weight of WT but not in *Csf1r^+/−^* mice was evident at day 8 of treatment. (**C–D**) Whole Animal Disease Activity Index for (WADI) WT (**C**) and *Csf1r^+/−^* (**D**) mice based on the modified criteria reported by Marshall *et al* (2007) [31] and described in the Methods. One WT mouse died at day 5 and one *Csf1r^+/−^* mouse died at day 7 whereas WT mice overall showed significantly elevated WADI compared to *Csf1r^+/−^* mice (*P<0.001, ANOVA with Bonferroni’s Multiple Comparison Test). Female mice of both genotypes show no observable clinical symptoms of inflammation (*data not shown*).

As pre-treatment of mice with neutralizing anti-CSF-1 antibodies protects them from DSS-induced colitis [31], we investigated whether loss of CSF-1R expression also afforded protection. Since *Csf1r^−/−^* FVB/NJ mice do not normally survive beyond 1 month of age, we queried whether loss of one *Csf1r* allele in *Csf1^+/−^* mice was sufficient. WT (*Csf1^+/+^*) and *Csf1^+/−^* mice were provided with water containing 2% w/v DSS *ad libitum* for 8 days and evaluated for inflammatory responses. While the weights of male WT male mice at day 8 had decreased significantly by ∼20% (mean +/− SEM; ***Day 0∶***31.42 g +/−0.90, ***Day 8∶***25.78 g +/−1.91; P<0.02; t-test) (**Figure 5A**), with one mouse dying at day 5, the weights of the *Csf1r* (mean +/− SEM; ***Day 0∶***32.00 g +/−0.71, ***Day 8∶***29.50 g +/−0/13; P = NS) (**Figure 5B**), with one mouse dying at day 7. In contrast, neither WT nor *Csf1r^+/−^* females exhibited significant DSS-induced weight loss and remained healthy after the 8-day treatment (*data not shown*). To further assess the effects of DSS treatment, we monitored the Whole Animal Disease Activity Index [31] as described in the Materials and Methods. Female mice of both genotypes showed no observable inflammation (*data not shown*). The most profound differences between the WT and *Csf1r^+/−^* male mice were noted at day 8 (**Figure 5C–D**), when male *Csf1r^+/−^* mice exhibited fewer symptoms compared to WT mice (p<0.001; ANOVA). Others have also reported that male mice are more susceptible to DSS-induced colitis, but not specifically in FVB/n mice [32], although this strain is differentially adversely affected in *mdr1a* mutant males [33].

**Figure 6:**
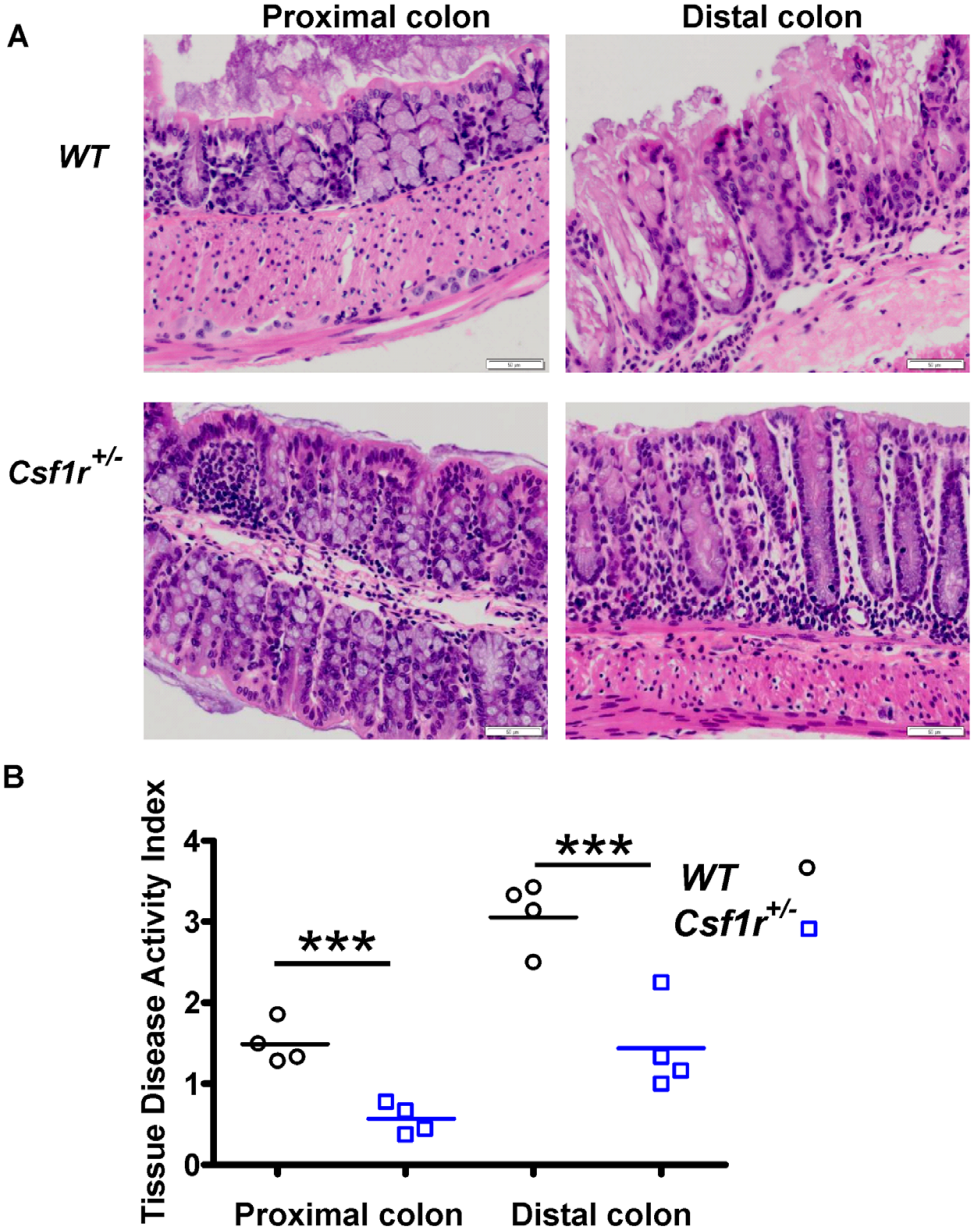
Male *Csf1r^+/−^* mice show less colonic epithelial damage compared to WT male mice. (A) Colons were separated into proximal and distal regions and stained with H & E, (Bar = 100 µm). (**B**) Crypt damage is more severe in the distal colon compared to proximal regions and is more severe in WT mice. Colonic crypt damage in each section (Tissue Disease Activity Index) was graded according to the modified criteria of Dieleman *et al* (1998) [34], as described in the Methods (n = 4, **P<0.01; ***P<0.001;data analyzed using two-tailed t-tests).

Further morphological evaluation of the SI and colons of WT and *Csf1r^+/−^* male mice failed to reveal significant differences in SI crypt or villi morphology, cell number or intestinal length (**Figure S4**). However, DSS treatment had pronounced effects on the colon as assessed using the modified criteria of Dieleman *et al* (1998) [34] (**Figure 6A**). Crypt damage was more severe in the distal colon compared to proximal regions and damage was observed in both WT and *Csf1r^+/−^* male mice. However, *Csf1r^+/−^* mice have significantly less crypt damage throughout the colon compared to WT mice **(**P<0.001 and 0.003; proximal and distal colon respectively; t-test) (**Figure 6B**). These data indicate that reduction of the *Csf1r* gene dosage has a dramatic impact on the inflammatory response mediating damage associated with exposure to DSS.

## Discussion

The maintenance of intestinal homeostasis requires the orchestration of many signaling pathways which may also be activated when the gastrointestinal tract is subjected to damage or stress [35], [36]. The recent discovery of the importance of the CSF-1R in SI homeostasis and differentiation [9] indicates that pathways downstream of this receptor could also be involved. The most obvious role of the CSF-1R in gut epithelium appears to be regulation of the development and maintenance of PC. However, CSF-1R deficiency is also associated with decreased *Lgr5*+ mRNA expression and decreased proliferation and altered cell fates of epithelial cells [9]. The discovery that Lgr5+ cells have the capacity to regenerate SI crypts and villi *in vitro* [37], that these cells are embedded among the PC in the crypts [37], that PC produce epithelial growth factors [30] and support Lgr5+ve intestinal stem cell propagation *in vitro* [37] and that there is local regulation of PC via CSF-1-expressing cells within the crypt [9], has drawn attention to the relatively long-lived PC population [38]. Indeed, PC appear to play an important role in responding to tissue damage and in activating the quiescent stem cell pool [39]. While PCs are not normally found in colon, because we observed decreased proliferation and altered cell fates of SI epithelial cells in CSF-1 - and CSF-1R -deficient mice, [9] we decided to examine the effects of CSF-1 and CSF-1R deficiency in normal colon and in mice with DSS-induced colitis. As discussed below, despite the absence of PC in colon, we observe defects in epithelial proliferation and differentiation that parallel those found in the SIs of CSF-1/CSF-1R-deficient mice.

In contrast to the localization of CSF-1R+ cells (PC) to the crypt base of the SI, in both the mouse [19] and human (**Figure 1**) colon the receptor is expressed throughout the crypt, despite the localization of Lgr5+ve cells in small numbers at the base of the colonic crypt [37]. It has been proposed that a mucous secreting non-goblet cell in the colonic crypt base is the PC equivalent cell [40] and recently it has been suggested that the cell surface antigen CD24 may mark the PC equivalent cells in the colon [30]. However, we found no significant difference in the expression of *CD24a* mRNA or of the mRNA encoding the PC product, R-spondin1, in *Csf1r^−/−^* and WT colonic crypts (**Figure S5**). (We could not detect CD24 by IHC or confocal microscopy in WT mouse colon despite its strong expression in the SI, *Akçora, et al, In Press*).

Previously we showed that expression of the cell surface CSF-1 isoform could completely rescue the CSF-1-deficient phenotype within the SI and that the CSF-1-reporter expressing cells were localized in the crypts adjacent to PC, suggesting that regulation of PC by CSF-1 was local, if not juxtacrine [9]. Recently, we specifically deleted the *Csf1r* gene in the mouse intestinal epithelium and found a reduction in PC, Lgr5 expression, cell proliferation and altered cell fate that phenocopied those observed in mice with the global *Csf1r* deletion (*Akçora, et al, In Press*). As PCs appear to be the only CSF-1R-expressing cells of the SI epithelium, these results are consistent with an aberrant regulation by PC of the proliferation and fate of epithelial cells resulting from disruption of the direct, local regulation of PCs by CSF-1. Thus it appears that it is through its action in PCs that the CSF-1R plays such a critical role in the SI. In colon, despite the lack of PCs, we have shown that, as in the SI, CSF-1R deficiency results in decreased cell proliferation, reduction in the expression of cell cycle genes and altered cell fate. Most importantly, as in SI, we have found that the expression of the mRNA for *Lgr5*, the most compelling stem cell molecular marker in the GI tract to date, is markedly reduced in the colonic crypts of *Csf1r^−/−^* compared with WT mice. Our failure to demonstrate robust CSF-1 stimulation of the proliferation of cells of two colonic epithelial cell lines, compared with their robust response to R spondin, suggests that, as in SI, epithelial cell proliferation is not directly regulated through the CSF-1R, despite the broader expression of the CSF-1R in colonic crypts. Thus these experiments demonstrate a critical role for the CSF-1R in the development of the colonic epithelium that does not appear to be a direct effect of regulation of the proliferation and differentiation of epithelial cells. In colon, it is possible that the CSF-1R is required for the development of cells with a function analogous to that of intestinal stem cell supporting PCs.

CSF-1 has been implicated in a range of inflammatory contexts [41]. A role for CSF-1 in GI inflammatory responses has been shown by the demonstration that administration of an anti-CSF-1 neutralizing antibody in mice is partially protective in DSS-induced colitis [31]. We used a genetic approach to examine the role of CSF-1R signaling in the same model system. We have shown that loss of one CSF-1R allele affords substantial protection from colon damage in male mice receiving a single challenge with DSS. In contrast, both WT and *Csf1r* ^+/−^ female mice were minimally affected by the same DSS treatment regime. Interestingly the same gender difference has been reported for colitis in man [42] and in there is indirect evidence that estrogen treatment might be protective in female patients with inflammatory bowel disease [43], [44]. In contrast to the protection we observed in male *Csf1r* ^+/−^ mice, heterozygous loss of *PPRAγ* gene in female mice exacerbated DSS-induced colitis [45]. The fact that *Csf1r^+/−^* mice are indistinguishable from WT litter-mates when unchallenged, but protected when stressed indicates the importance of *Csf1r* gene dosage in DSS-induced colitis. Humans with hypomorphic mutations that dampen CSF-1R signaling might be protected from IBD and, as has been suggested by others [31], therapies inhibiting CSF-1 or CSF-1R may be beneficial in the treatment of this disease. However, additional studies are needed to determine whether DSS treatment affects the colon directly, or if the acute damage response is mediated by macrophages.

CSF-1 is the primary regulator of monocyte proliferation and differentiation [46], but more broadly it has also been implicated in trophoblastic implantation [47] and mammary gland development particularly during pregnancy [48]. The CSF-1R is expressed in normal intestinal [49] and upper airway [50] epithelia and lung, ovarian, breast and prostate cancers, or epithelial cell lines derived from them [51], [52], [53], [54]. Furthermore normal and adenomatous polyps and colon tumors express the CSF-1R [52], [53] and its expression is evident in the lamina propria macrophages of the SI [55]. CSF-1R signaling also plays an augmenting role in the expansion of hematopoietic stem and progenitor cells [14], [56], [57], an indispensible role in osteoclastogenesis [58] and regulates both microglia and neural progenitors in brain [59]. Our previous observations [9] in SI, coupled with these studies in colon, showing that multiple epithelial lineages are influenced by CSF-1R ablation, clearly indicate that this receptor plays an important role in the development and function of the gastrointestinal tract.

## Methods

### Human Colon Crypts

Normal human mucosa was obtained from a surgical resection for colon cancer as tissue adjacent, but separate from the tumor, with institutional human ethics approval. Crypts were released as described elsewhere [60] and subjected to confocal microscopy as reported previously [19] using anti-CD115/c-FMS/CSF-1R (abm-77 Rat anti-human monoclonal RDI-Fitzgerald) at 1∶250 and secondary anti-Rat FITC antibody.

### Mice

*Csf1* ^op/+^ [10] and *Csf1r^−/^* ^+^ [15] mice, backcrossed on the FVB/NJ background for at least 10 generations [16], were used to generate homozygous mutant and WT (+/+) control mice. Mice were housed under SPF conditions and all experimentation was carried out with approval of the Peter MacCallum Cancer Centre institutional animal ethic committee (Project #E389).

### Histochemistry, Immunohistochemistry and Immunofluorescence

Post-natal day 14 mouse pups were perfused at atmospheric pressure with periodate-lysine-2% paraformaldehyde-0.05% glutaraldehyde, pH 7.4 (PLPG) [61], their intestines from the anus to stomach removed and opened longitudinally by incision along the length of the intestine, the contents removed by rinsing in PBS and the intestines fixed in PLPG overnight, prior to immersion in 70% ethanol. Paraffin embedding of the tissues was arranged such that tissue orientation could be determined. Sections were treated with periodic acid, then stained in Schiff’s reagent (0.5% pararosanaline, 1% sodium metabisulfite; PAS staining) and counterstained with hematoxylin. Some sections were also stained with Alcian Blue. The mouse anti-PCNA antibody, PC-10 (1∶800) (Dako, #M0879) was used to identify PCNA followed by processing with the Dako Envison+ mouse detection kit and photographed using a Olympus Bx51 upright microscope. For Myb and hematoxylin and eosin (H & E) staining, colon sections were fixed in methacarn (60% methanol, 30% chloroform, 10% acetic acid) for 2 h and transferred to 70% ethanol, embedded, sectioned and stained with H & E. Full crypts, exposing a lumen and identifiable base, were scored. Crypt cells per 40–50 longitudinal sections were scored as previously described [62]. Sample groups were subjected to one-way analysis of variance (ANOVA) using Origin^R^ software. c-Myb was visualized using Mab1.1 and processed as described previously [63] using antigen retrieval by boiling slides in 1 mM EDTA in a pressure cooker for 3 min. Rabbit anti-Chromogranin A was used at 1∶100 (SC-13090-Santa Cruz) and anti-Phospho-histone 3 at a final titer of 1∶200 (Upstate Biotech) following citrate buffer antigen retrieval. Donkey anti-goat-HRP at 1∶250 (Santa Cruz) was used as a secondary antibody. Crypt cell counting was performed on longitudinal sections, blinded for genotype where nuclei were used to identify each cell. Crypts were identified at 100X magnification, whereby the lumen could be seen to traverse the crypt, then counted at 400X magnification. At least 40 crypts were examined per genotype with 3–5 mice per genotype.

### Dextran Sulfate Sodium (DSS) Studies

Female and male FVB/n mice aged from 8–10 weeks were fed water with 2% w/v DSS *ad libitum* for 8 days and observed for inflammatory responses as well as changes in body weight. Clinical symptoms were assessed based on the modified criteria reported by Marshall *et al.,* 2007 [31] to define the Animal Disease Activity Index, in which a higher score correlates with increasing severity: 0 = Healthy; 1 = Appearance of diarrhoea; 2 = Sign of fecal blood, 3 = Bloody diarrhoea; 4 = Bleeding from the anus. For histology, colons were separated into proximal and distal regions and the regions were then further divided into 600 µm sections. Colonic crypt damage in each section was graded according to the modified criteria published by Dieleman *et al* (1998) [34], which we describe as the Tissue Disease Activity Index: 0 = No damage; 1 = Basal 1/3 damaged; 2 = Basal 2/3 damaged; 3 = Only surface epithelium intact; 4 = Entire crypt and epithelium lost.

### Quantitative RT-PCR Analysis of Crypt Epithelium RNA

Crypt epithelium was prepared and its purity confirmed as described [64]. Real-time RT-PCR reactions were conducted on genomic DNA-depleted RNA using the Bio-Rad iQ-5 i-cycler system (Bio-Rad Laboratories, CA) and the appropriate primers. Gene expression was normalized to *gapdh.*

### Cell Culture

YAMC [26] and IM-5 [19] cells are grown in RPMI-1640 plus HEPES medium with 10% FCS (fetal calf serum). For the growth factor stimulated proliferation studies, cells were plated in 12 well plates at low density and allowed to attach for 24 hr in FCS-containing medium. The wells were washed three times with PBS and the cells incubated for an additional for 24 hr in FCS-free medium, followed by further incubation in FCS-free medium containing different concentrations of pregnant mouse uterine extract (PMUE), as a source of CSF-1 [19], or purified growth factors. Viable cells were scored at 3–5 days post-growth factor addition by incubation in MTT (3-(4,5-Dimethylthiazol-2-yl)-2,5-diphenyl tetrazolium bromide), followed by cell lysis and reading of the optical density at 570 nm. The growth factors, recombinant murine CSF-1 (Peprotech), murine interleukin 34 (IL-34), Wnt3a and R-Spondin (500 ng/ml; R&D Systems), were diluted to their final working concentrations in PBS immediately prior to addition to cell cultures. For mRNA analyses, cells were washed with PBS and then lysed in Tryzol. RNAse-free DNase1-treated total RNA was then processed for RT-PCR. List of primers used in Q-RT-PCR analyses is provided in **Table 1**.

**Table 1:** List of primers used in Q-RT-PCR analyses.

Gene	Forward Primer	Reverse Primer
c-myc	5′-AAGGCCCCCAAGGTAGTGA-3′	5′-TCCATTCAAGCAGACGAGCA-3′
c-myb	5′-AATTATCTGCCCAACCGG-3′	5′- AGACCAACGCTTCGGACC-3′
Csf1r	5′-CCTCCTCTGGTCCTGCTG-3′	5′-CATTCCACACTGCCATTGC-3′
cyclinD1	5′-AGGCTACAGAAGAGTATTTATGGGAAA-3′	5′-TGCGTTTGAATCAAGGGAGAT-3′
lgr5	5′-CAAGCCATGACCTTGGCCCTG-3′	5′-TTTCCCAGGGAGTGGATTCTATT-3′
c-fos	5′-CCGATGACCTTGGCTTCC-3′	5′-TGCTGATGCTCTTGACTGG-3′
c-jun	5′-GCAGACAGACAGACAGAC-3′	5′-GAAGACAAACGGATGAACAG-3′
ets-2	5′-GATCGCGCACTTCCGCTCTC-3′	5′-GATCGAGAGCGGAAGTGCGC-3′
R-spondin-1	5′-CAAGGGCAAGAGACAGAG-3′	5′-TCCAGCAGAATGAAGAGC-3′
CD24a	5′ GGCAACCACAAGTCCAATG-3′	5′-AACTCCAGCAGATTCAATAGC-3′
Gapdh	5′-CAACTACATGGTCTACATGTTCCAGTATG-3′	5′-CTCCCTAGGCCCCTCCTGTTATTAT-3′

### Cytokine Array

RayBio^R^ Cytokine antibody Array 3 was used to assess the presence of CSF-1 and other factors present in PMUE according to manufacturer instructions.

### Statistics

Data were analyzed using GraphPad Prism5 statistical package where p<0.05 was considered to be statistically significant.

## Supporting Information

Figure S1 **Alcian blue (AB) staining shows increased acidic mucin production in** ***Csf1r^−/−^*** **and** ***Csf1^op/op^*** **colonic epithelium.** Similar to, but more extensive than observed with PAS staining (**Figure 1**), AB staining shows aberrant goblet cell localization and mucin deposition in both the proximal and distal colon. Mucin deposition was most pronounced in the *Csf1r^−/−^* colonic epithelium. Bar = 50 µm.
(PDF)

Figure S2 **PMUE-stimulation of immortalized colonic epithelial cells YAMC cells following serum starvation.** (A) Five×10^4^ YAMC cells were cultured in fetal calf serum-free media with and without increasing amounts of PMUE (a source of CSF-1) prior to assessment of viable cell numbers by MTT assay at day 5. ***Upper panel:*** Representative images of wells incubated without or with the indicated concentrations of PMUE. ***Lower panel:*** Quantitation of data from multiple plates (n = 4, *P<0.05; **0.01; ***0.001; ANOVA with Bonferroni’s multiple comparison Testing). **(B)** Time course of Immorto-5 (IM-5) cells Erk1/2 phosphorylation status in response to PMUE. Cytosolic fractions of cells grown with fetal calf serum (FCS), or serum-starved, or serum-starved and then incubated with 5 µl/ml PMUE for the indicated times were subjected to SDS-PAGE and western blotted for phospho-ERK 1/2 and total ERK 1/2. **(C)** Cytokine antibody arrays show that CSF-1 is the predominant, but not the only growth factor/cytokine in PMUE (red ellipse). The next most abundant factors identified were IGFBP-3 and MIP-2 (black rectangles). **(D–E)** IM-5 (like YAMC, *data not shown*) cells show robust proliferation by MTT assay in response to PMUE but and slight stimulation by purified the CSF-1R ligands, CSF-1 or IL-34. **(F)** Immorto-5 cells respond strongly to R-spondin in the presence or absence of CSF-1, while Wnt3a has no stimulatory effect alone or in combination with R-spondin or CSF-1, (*P<0.05; **0.01. *P<0.001, analysed using one-tailed t-tests).
(PDF)

Figure S3 **Cell cycle and immediate gene expression induction in colonic epithelial cells following PMUE stimulation.** IM-5 cells were cultured in the presence of FCS (+FCS), or serum-starved (-FCS), or serum-starved and incubated with 5 µl/ml PMUE for the indicated times prior to extraction of RNA for analysis of gene expression by qRT-PCR. Results show induction of immediate early genes (*ets-2 & c-Jun*) and cell cycle genes (*c-myc, c-myb & cyclinD1*) following PMUE stimulation, (Means ± SEM, 6 replicates. **P<0.01; one way ANOVA with Bonferroni’s multiple comparison testing).
(PDF)

Figure S4 **Absence of obvious differences between male FVB/NJ** ***Csf1r^+/−^*** **and WT small intestines following DSS-induced colitis.** As it has been reported that an increase in villus height and crypt depth may occur in response to DSS-induced colitis [65], the number of cell nuclei in the small intestinal villus and crypt of male mice was determined (25 crypt & villi per region, n = 4). No morphological (duodenum, jejunum or ileum) or numerically significant differences in cells per crypts ***(bottom left panel)*** or villi ***(bottom middle panel)*** between WT and *Csf1r^+/−^* mice was observed. In addition, no significant differences in intestinal length between the DSS-treated WT and *Csf1r^+/−^* mice were detected in small intestines or colons ***(bottom right panel)***. Images are representative H&E stained section of small intestine, (Bar = 100 µm).
(PDF)

Figure S5 ***CD24a*** **and** ***R-spondin*** **mRNA expression are not significantly altered in** ***Csf1r^−/−^*** **colonic crypts.** (A) Absence of *Csf1r* mRNA in *Csf1r*−/− crypts. No significant change in *CD24a* mRNA **(B)** or *R-spondin-1* mRNA **(C)** expression in *Csf1r^−/−^* compared with WT crypts was observed, (Means ± SEM, 4 replicates. **P<0.05; one-tailed t-test).
(PDF)
