# Supplementary figures and images for: CSF-1 Receptor-Dependent Colon Development, Homeostasis and Inflammatory Stress Response
Source: PLoS One. 2013 Feb 22;8(2):e56951. doi: 10.1371/journal.pone.0056951 (PMC3579891; doi:10.1371/journal.pone.0056951)

## Supplementary Figure 1

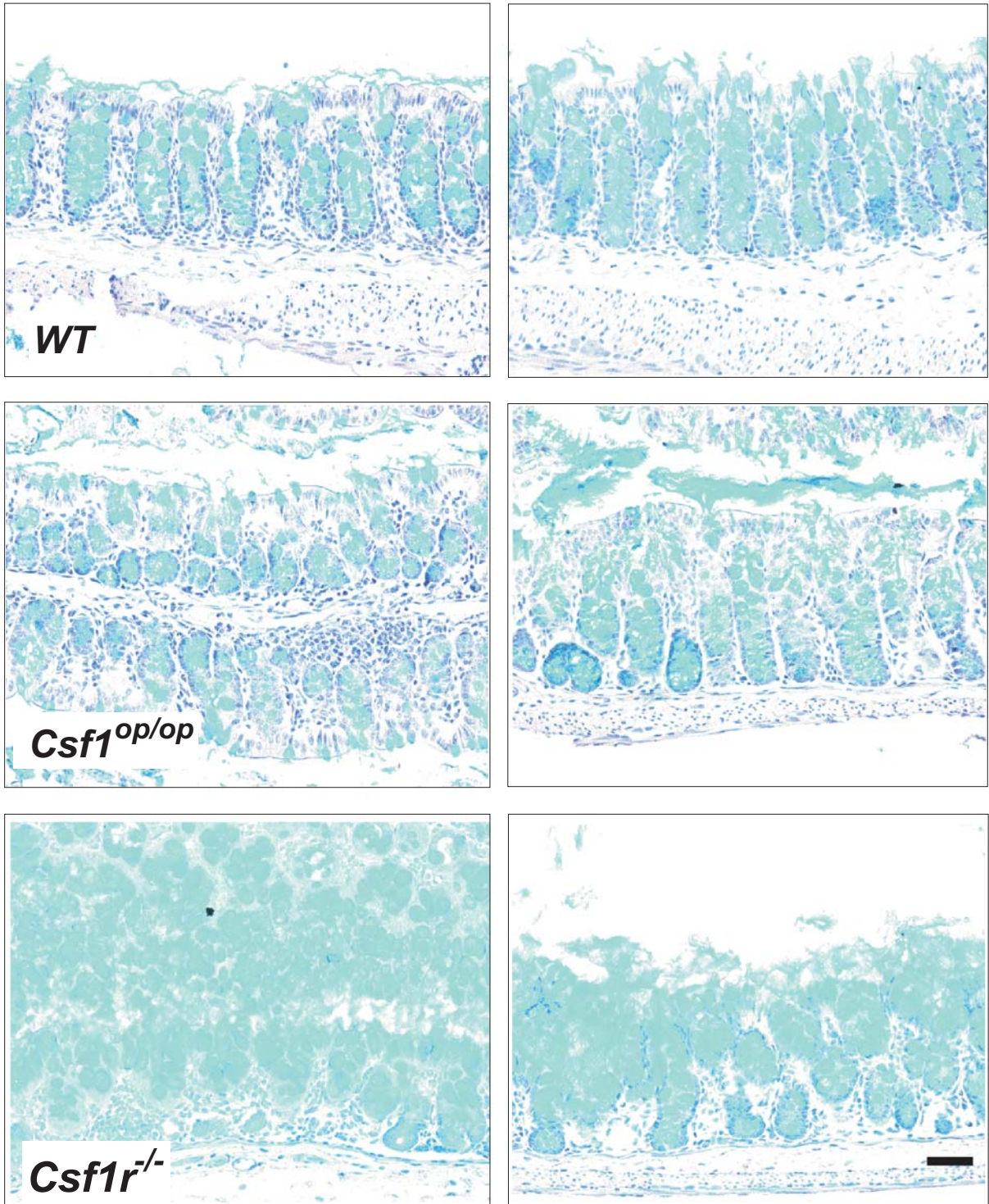

Supplement: Figure S1 — Alcian blue (AB) staining shows increased acidic mucin production in Csf1r−/− and Csf1op/op colonic epithelium. Similar to, but more extensive than observed with PAS staining ( Figure 1 ), AB staining shows aberrant goblet cell localization and mucin deposition in both the proximal and distal colon. Mucin deposition was most pronounced in the Csf1r−/− colonic epithelium. Bar = 50 µm. (PDF) [file pone.0056951.s001.pdf]

# Supplementary Figure 2

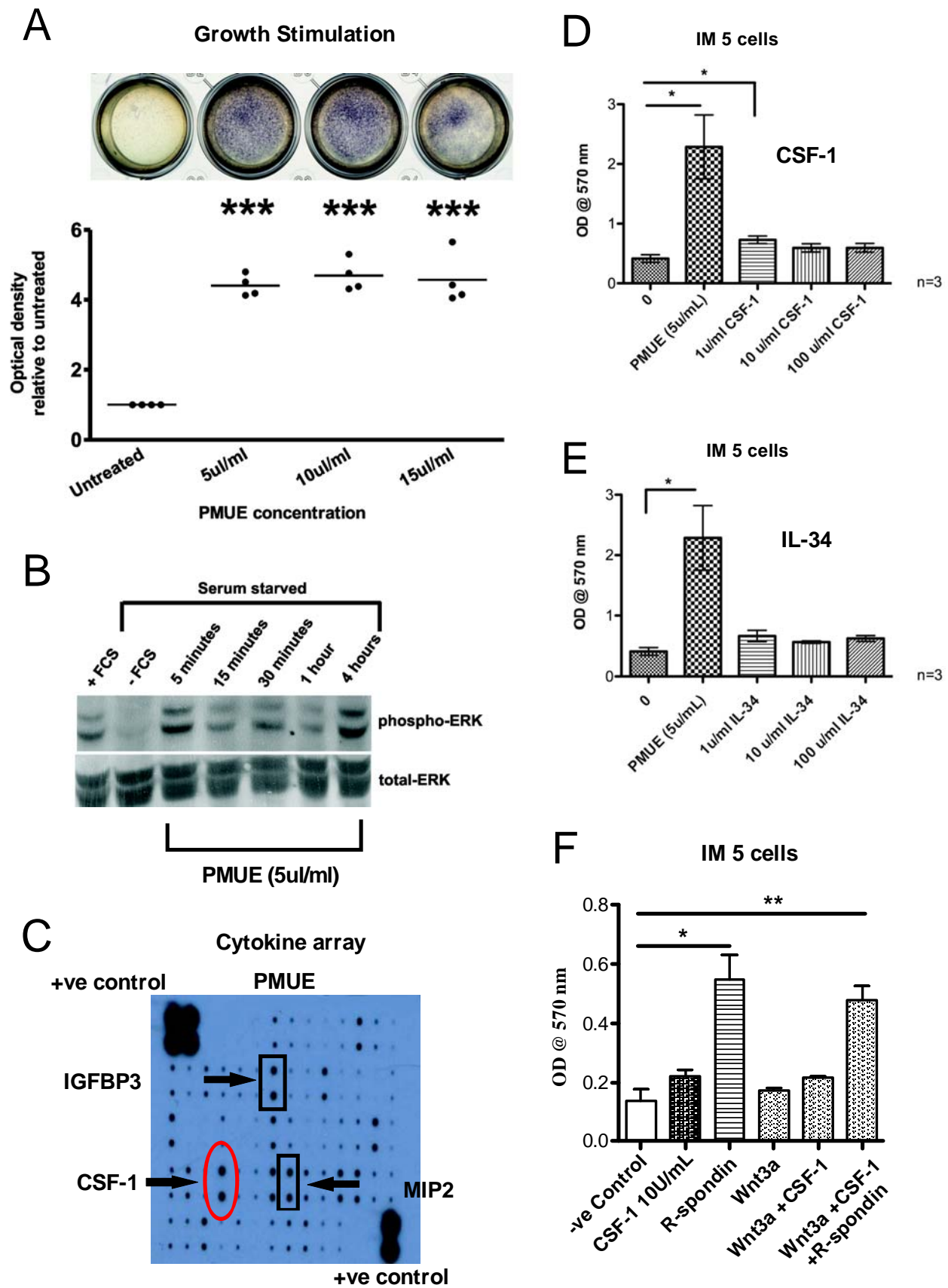

Supplement: Figure S2 — PMUE-stimulation of immortalized colonic epithelial cells YAMC cells following serum starvation. (A) Five×104 YAMC cells were cultured in fetal calf serum-free media with and without increasing amounts of PMUE (a source of CSF-1) prior to assessment of viable cell numbers by MTT assay at day 5. Upper panel: Representative images of wells incubated without or with the indicated concentrations of PMUE. Lower panel: Quantitation of data from multiple plates (n = 4, *P<0.05; **0.01; ***0.001; ANOVA with Bonferroni’s multiple comparison Testing). (B) Time course of Immorto-5 (IM-5) cells Erk1/2 phosphorylation status in response to PMUE. Cytosolic fractions of cells grown with fetal calf serum (FCS), or serum-starved, or serum-starved and then incubated with 5 µl/ml PMUE for the indicated times were subjected to SDS-PAGE and western blotted for phospho-ERK 1/2 and total ERK 1/2. (C) Cytokine antibody arrays show that CSF-1 is the predominant, but not the only growth factor/cytokine in PMUE (red ellipse). The next most abundant factors identified were IGFBP-3 and MIP-2 (black rectangles). (D–E) IM-5 (like YAMC, data not shown) cells show robust proliferation by MTT assay in response to PMUE but and slight stimulation by purified the CSF-1R ligands, CSF-1 or IL-34. (F) Immorto-5 cells respond strongly to R-spondin in the presence or absence of CSF-1, while Wnt3a has no stimulatory effect alone or in combination with R-spondin or CSF-1, (*P<0.05; **0.01. *P<0.001, analysed using one-tailed t-tests). (PDF) [file pone.0056951.s002.pdf]

## Supplementary Figure 3

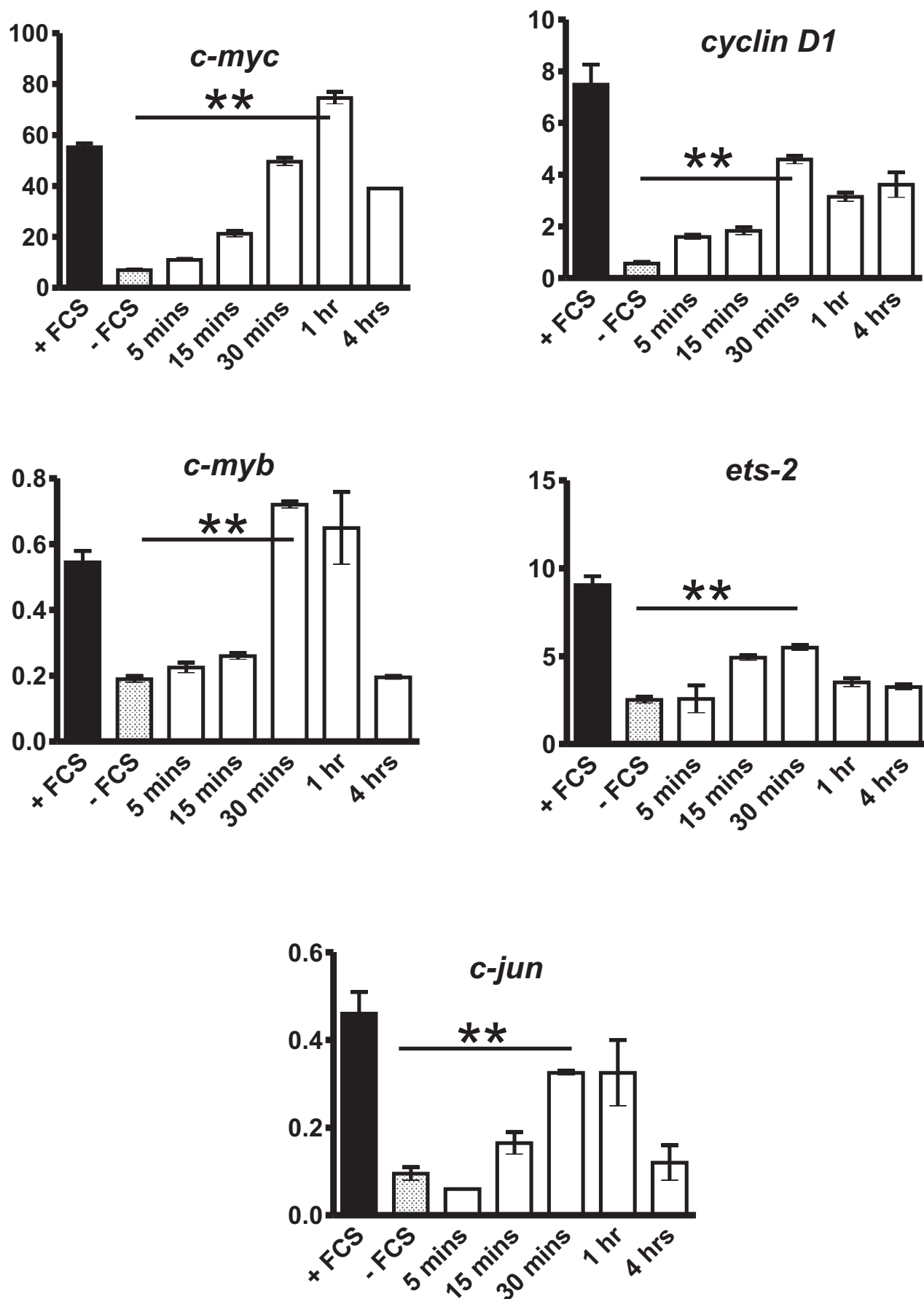

Supplement: Figure S3 — Cell cycle and immediate gene expression induction in colonic epithelial cells following PMUE stimulation. IM-5 cells were cultured in the presence of FCS (+FCS), or serum-starved (-FCS), or serum-starved and incubated with 5 µl/ml PMUE for the indicated times prior to extraction of RNA for analysis of gene expression by qRT-PCR. Results show induction of immediate early genes (ets-2 & c-Jun) and cell cycle genes (c-myc, c-myb & cyclinD1) following PMUE stimulation, (Means ± SEM, 6 replicates. **P<0.01; one way ANOVA with Bonferroni’s multiple comparison testing). (PDF) [file pone.0056951.s003.pdf]

Supplementary Figure 4

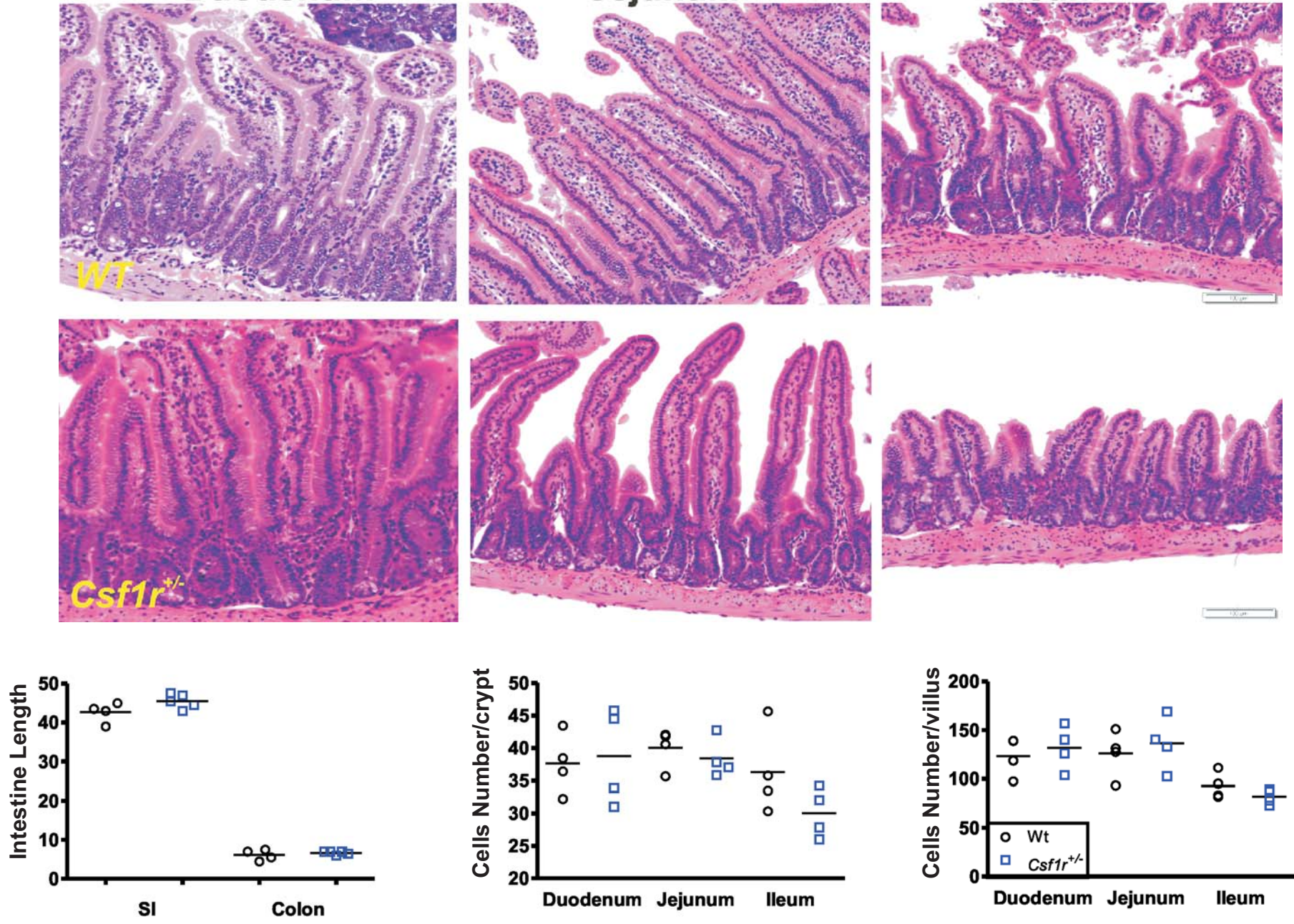

Supplement: Figure S4 — Absence of obvious differences between male FVB/NJ Csf1r+/− and WT small intestines following DSS-induced colitis. As it has been reported that an increase in villus height and crypt depth may occur in response to DSS-induced colitis [65], the number of cell nuclei in the small intestinal villus and crypt of male mice was determined (25 crypt & villi per region, n = 4). No morphological (duodenum, jejunum or ileum) or numerically significant differences in cells per crypts (bottom left panel) or villi (bottom middle panel) between WT and Csf1r+/− mice was observed. In addition, no significant differences in intestinal length between the DSS-treated WT and Csf1r+/− mice were detected in small intestines or colons (bottom right panel). Images are representative H&E stained section of small intestine, (Bar = 100 µm). (PDF) [file pone.0056951.s004.pdf]

## Supplementary Figure 5

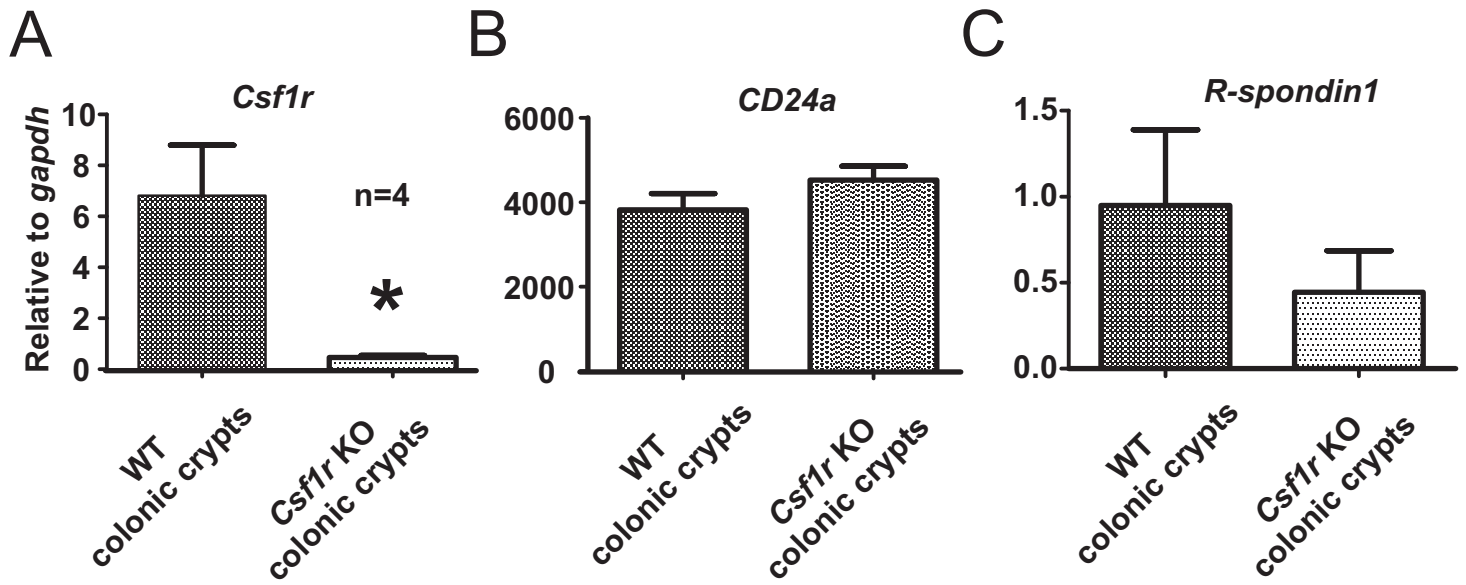

Supplement: Figure S5 — CD24a and R-spondin mRNA expression are not significantly altered in Csf1r−/− colonic crypts. (A) Absence of Csf1r mRNA in Csf1r−/− crypts. No significant change in CD24a mRNA (B) or R-spondin-1 mRNA (C) expression in Csf1r−/− compared with WT crypts was observed, (Means ± SEM, 4 replicates. **P<0.05; one-tailed t-test). (PDF) [file pone.0056951.s005.pdf]
